# Not All Sacral Wounds Are Sacral Decubitus Ulcers: A Case Report

**DOI:** 10.5811/cpcem.57440

**Published:** 2023-11-08

**Authors:** Forrest Lindsay-McGinn, Cory Munden

**Affiliations:** * Kaiser Permanente Vacaville Medical Center, Department of Emergency Medicine, Vacaville, California; † Hospital of the University of Pennsylvania, Department of Emergency Medicine, Philadelphia, Pennsylvania

**Keywords:** *herpes zoster*, *sacral wounds*, *immunocompromised*, *disseminated*, *case report*

## Abstract

**Introduction:**

Sacral wounds are commonly seen in the emergency department and typically get diagnosed as a pressure ulcer of varying stage. However, other disease processes and infections can affect the sacrum.

**Case Report:**

Presented here is the case of an evolving sacral wound in a 70-year-old, immunocompromised woman that was eventually found to be localized herpes zoster and later became disseminated.

**Conclusion:**

This case demonstrates the need for a broad differential diagnosis for sacral wounds that include atypical presentations for herpes zoster or herpes simplex virus. We discuss the guidelines for treatment and the classification of localized vs disseminated herpes zoster.

CPC-EM Capsule What do we already know about this clinical entity?

*Herpes zoster is a viral infection that affects the skin and nervous system in a dermatomal distribution and is frequently diagnosed in the emergency department.*

What makes this presentation of disease reportable?

*This is an atypical presentation of herpes zoster, which appeared similar to a sacral decubitus ulcer and evolved into disseminated herpes zoster.*

What is the major learning point?

*Herpes zoster can present atypically, especially in immunocompromised patients. These patients have an increased likelihood of having disseminated herpes zoster.*

How might this improve emergency medicine practice?

*Keep a wide differential for skin wounds, especially atypical sacral wounds, and send a herpes simplex virus and varicella-zoster virus swab if concerned.*

## INTRODUCTION

Herpes zoster is a viral infection that commonly affects the skin and nervous system and occurs in more than 1.2 million individuals annually.^1^ It appears in many areas of the body with the most common dermatomes affected being thoracic (55%), cervical (20%), trigeminal including ophthalmic (15%), and lumbosacral (11%).^2^ It commonly presents in one or two adjacent dermatomes and usually does not cross midline; however, there have been limited reports of bilateral herpes zoster.^3^ Disseminated zoster is typically described as 20 or more lesions beyond the primary or adjacent dermatome.^2^ Common complications of herpes zoster include: ophthalmic involvement (possible vision loss), bacterial superinfection of the lesions (*Staphylococcus aureus* and less commonly *Streptococcus pyogenes*), cranial and peripheral nerve palsies, pneumonitis, hepatitis, or meningoencephalitis, among others.^4^ This case report illustrates the need for a broad differential when evaluating a sacral wound to prevent misdiagnosis and initiate prompt treatment and care of the patient.

## CASE REPORT

A 70-year-old woman with lupus complicated by shrinking lung syndrome and chronic respiratory failure, chronic anemia, ovarian cancer in remission, type 2 diabetes mellitus, hypertension, and necrotizing fasciitis, currently on hydroxychloroquine and mycophenolate mofetil, presented to the emergency department (ED) for symptomatic anemia. On initial lab work, the patient had a glucose of 139 milligrams per deciliter (mg/dL) (reference range 70–99 mg/dL), hemoglobin of 7.0 grams (g)/dL (13.5–17.5 g/dL), and a white blood cell count of 7.6 thousand per microliter/μL (4.0–11.0 thousand/μL). The patient also reported an evolving “bruise” to her sacral region that had developed over the prior seven days. She described a burning sensation for the first three days, which had resolved. The patient was fully ambulatory, without periods of immobilization. On exam of her sacral area (Image), there was a 5 centimeter area of shallow ulceration with scalloped gray borders, dark purple erythema, and an erythematous base with satellite lesions extending caudally from the wound. The ulcer was dry and nontender to touch. Dermatology was consulted in the ED due to concern for vasculitis.

**Image.**
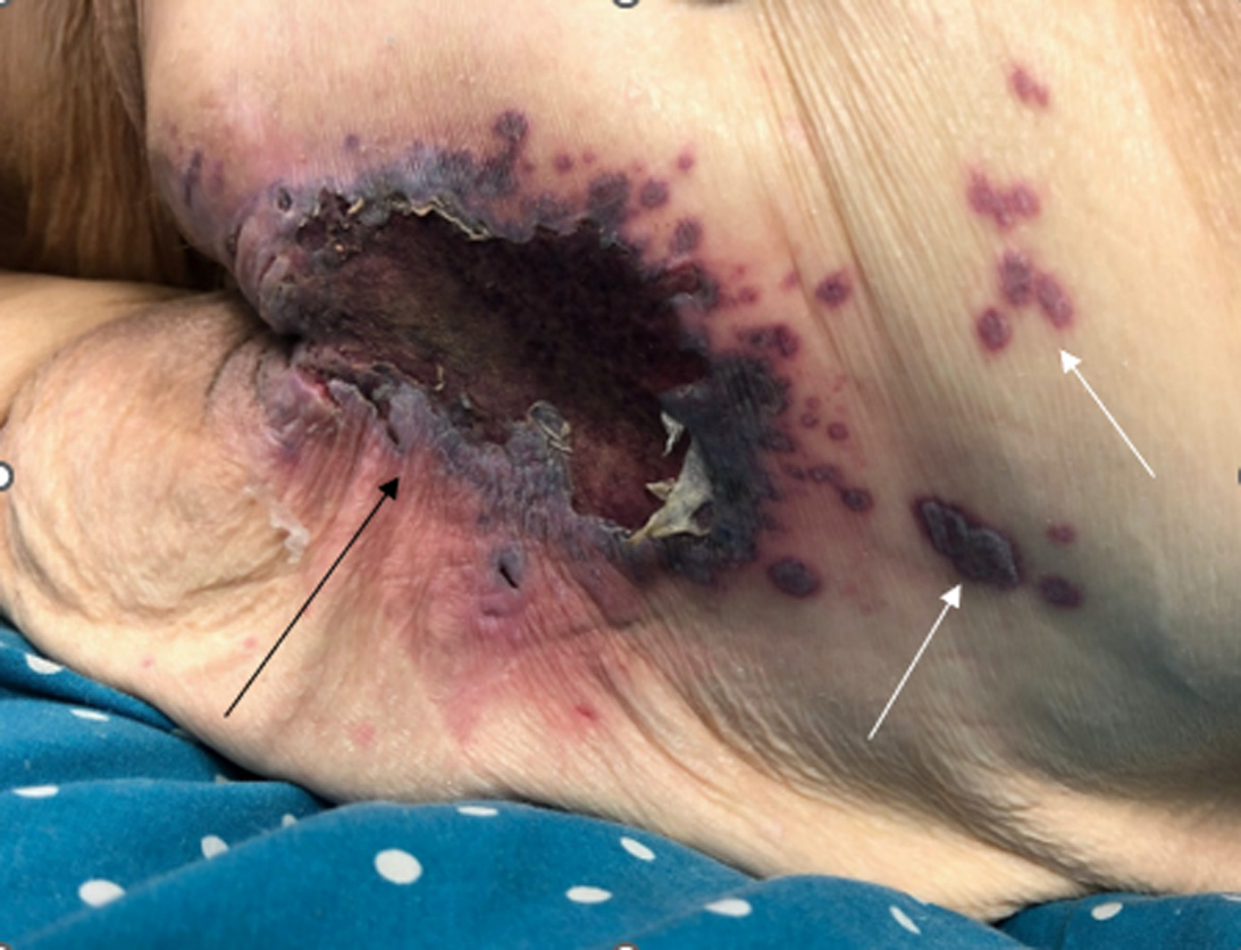
Sacral wound (black arrow) found on the patient, with satellite lesions (white arrows) superior to sacral wound.

The dermatology team obtained further context about the lesion—there was no prodrome prior to the lesion developing, and the patient had a prior history of cold sores, most recently three months prior. The patient also had photographs showing vesicular lesions in the satellite lesions of the wound during the first several days. The dermatology team was concerned for herpes simplex virus (HSV) or herpes zoster virus and sent a swab of the wound for varicella-zoster virus (VZV) and HSV polymerase chain reaction test. This patient’s rash was noted to cross midline but was localized to two adjacent dermatomes with only two satellite lesions; thus, the rash was considered localized and not disseminated. The patient was started on valacyclovir 1,000 milligrams three times daily for the next seven days and mupirocin 2% ointment and discharged to home.

The patient’s sacral wound swab tested positive for VZV and negative for HSV. She returned four days later with worsening pain at the sacral wound and over 20 new vesiculopustular lesions to her nose, lower back, abdomen, and posterior oropharynx. She was admitted for disseminated herpes zoster virus at that time and was started on intravenous acyclovir. Her hospital course showed improvement in her lesions with crusting, and she was transitioned to oral valacyclovir and discharged to home on hospital day seven to finish her course of oral valacyclovir and to start lifelong prophylactic dosing of valacyclovir.

## DISCUSSION

In a patient with a sacral wound, the leading differential is a pressure-induced sacral decubitus ulcer of varying stage as these are commonly seen in the ED. However, this patient was fully ambulatory and had no prolonged periods of immobilization, making a pressure-induced wound exceedingly unlikely; ultimately, this led to a dermatology consult. The differential for sacral wounds should include cellulitis, contact dermatitis, ischemic ulcer, venous ulcer, necrotizing fasciitis, hypertensive ulcers, vasculitis, HSV, varicella-zoster virus virus, and candidiasis among others.

Herpes zoster can also present atypically in immunocompromised individuals. One presentation, hemorrhagic herpes zoster, is described as a purpuric or ecchymotic base. It is an atypical presentation and typically occurs in patients who are immunosuppressed, thrombocytopenic, or coagulopathic.^5^ There are six types of herpes zoster documented, which can cloud diagnostic capability: bullous; verrucous-crusted; hemorrhagic, ulcerative-necrotic-gangrenous, disseminated (or varicelliform), and double herpes zoster (which involves at least one dermatome on both sides of the body and is not symmetrical in appearance).^6^

In addition, HSV may develop in a similar distribution to herpes zoster and mimic zoster (zosteriform herpes simplex); it frequently occurs in the face or genital/buttock regions. Therefore, it is important to send both HSV and VZV swabs for patients with suspicion of herpes zoster or herpes simplex.

## CONCLUSION

This case demonstrates the need for a broad differential diagnosis for sacral wounds that include atypical presentations for herpes zoster and herpes simplex virus, as a missed diagnosis could result in delay to appropriate treatment. In cases of herpes zoster, it is essential that the clinicia focus immediate efforts on determining whether herpes zoster is localized or disseminated and whether the patient is immunocompetent or immunocompromised. Per US Centers for Disease Control and Prevention guidelines, when treating an immunocompetent patient with localized herpes zoster, no airborne or contact precautions are needed.

If the patient is immunocompromised and has localized herpes zoster the infection should be considered airborne, and contact and airborne precautions should be taken until disseminated infection is ruled out.^7^

Herpes zoster is considered disseminated when there are 20 or more vesicles outside the primary or adjacent dermatomes.^2^ Disseminated herpes zoster occurs in about 2% of the general population and up to 15–30% in immunocompromised hosts.^8^ Finally, if herpes zoster is disseminated in either an immunocompetent or immunocompromised patient, airborne and contact precautions should be taken until the lesions are crusted.^7^
